# Identification and validation of PGLS as a metabolic target for early screening and prognostic monitoring of gastric cancer

**DOI:** 10.1002/jcla.24189

**Published:** 2021-12-24

**Authors:** Xiaoxia Yuan, Yang Xiao, Yaomin Luo, Chen Wei, Jiaxin Wang, Jinglin Huang, Weiliang Liao, Shenjie Song, Zhen Jiang

**Affiliations:** ^1^ Department of Biochemistry and Molecular Biology School of Preclinical Medicine North Sichuan Medical College Nanchong China

**Keywords:** 6‐phosphogluconolactonase, gastric cancer, iTRAQ

## Abstract

**Background:**

Gastric cancer is the third leading cause of cancer‐related death in the world. The purpose of the present study is to investigate the expression and prognostic significance of 6‐phosphogluconolactonase (PGLS) in gastric cancer.

**Methods:**

The protein extracted from a panel of four pairs of gastric cancer tissues and adjacent tissues, labeled with iTRAQ (8‐plex) reagents, and followed by LC‐ESI‐MS/MS. The expressions of proteins were further validated by immunohistochemistry analysis. The expression levels of mRNA were analyzed and validated in the Oncomine database. The correlations of PGLS with prognostic outcomes were evaluated with Kaplan‐Meier plotter database.

**Results:**

The present study found that PGLS was significantly up‐regulated in gastric cancer by using iTRAQ‐based proteomics and immunohistochemistry analysis. The sensitivity of PGLS in gastric cancer was 72.9%. The high expression of PGLS was significantly correlated with TNM staging in gastric cancer (*p *= 0.02). The overexpression of PGLS predicts worse overall survival (OS) and post‐progression survival (PPS) for gastric cancer (OS, HR = 1.48, *p *= 2.1e‐05; PPS, HR = 1.35, *p *= 0.015). Specifically, the high PGLS expression predicts poor OS, PPS in male gastric cancer patients, in patients with lymph node metastasis and in patients with Her‐2 (‐).

**Conclusions:**

These findings suggested that PGLS was aberrantly expressed in gastric cancer and predicts poor overall survival, post‐progression survival for gastric cancer patients. The present study collectively supported that PGLS is an important target for early determining and follow‐up monitoring for gastric cancer.

## Graphical abstract

Gastric cancer patients with high 6‐phosphogluconolactonase (PGLS) expression presented poor overall survival (OS) and post‐progression survival (PPS) and first progression (FP). (A), Overall survival curves. (B), Post‐progression survival curves. (C), First progression curves.

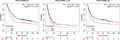

## 1 INTRODUCTION

Gastric cancer is the third leading cause of cancer‐related death in the world, which accounted for more than 784,000 deaths in 2018. There is often a long precancerous disease period about 44 months before the formation of gastric cancer. There are numerous risk factors for gastric cancer that have been identified, such as *H. pylori* infection, age, diets high salt, and low in vitamin.^1^

Although the advancement of diagnostic and therapeutic methods has been made in recent years, a number of patients remain have poor prognosis partially because it was almost advanced stage when patients were diagnosed with gastric cancer.^2^, ^3^ Previous studies have demonstrated that gastric cancer patients diagnosed in early stage have a survival rate of up to 61%. However, if they were diagnosed in advanced stage, they only have a 5‐year survival rate of 24%.^4^ In order to screen early‐stage gastric cancer patients, several methods have been widely used in clinical practice, such as *H. pylori* infection testing, the serum pepsinogen test, and the gastrin 17 test. In addition, serum CA‐199 and carcinoembryonic antigen (CEA) were widely used for screening early gastric cancer. However, these methods and biomarkers yield low sensitivity and specificity.^5^ Therefore, the identification of novel diagnostic biomarkers which can sensitively diagnose primary tumor and metastatic cancer is urgently needed.

Cancer metabolism is a complex process where the cancer cells can acquire specific traits that enable them to survive from extremely microenvironments. A number of metabolic enzymes which were found to be aberrantly expressed in cancer cells have profound impact on tumor progression and metastasis.^6^, ^7^ The reaction catalyzed by these metabolic enzymes is closely involved in tumor oxidation reduction and microenvironments which promote the cancer cell proliferation, survival, angiogenesis, and evasion of host immune response, etc.^8^, ^9^ Hence, studying the key metabolic enzymes expressed by the tumor may yield a new range of potential biomarkers and therapeutic targets.

Proteomics approaches are powerful tools for identifying biomarkers in tissue specimens of malignant tumors. Traditional 2‐DE‐based proteomic yields low sensitivity and specificity outcome. Isobaric tags for relative and absolute quantification (iTRAQ) technology and LC‐ESI‐MS/MS were currently the most widely used methods for high‐throughput protein quantification.^10^, ^11^, ^12^

In this study, we performed iTRAQ‐based LC‐ESI‐MS/MS to analyze the gastric cancer tissues proteome compared with adjacent cancer tissues. We aim to identify a range of novel metabolic signatures in gastric cancer and to evaluate the specificity and sensitivity of these candidates for gastric cancer diagnosis.

## 2 MATERIALS AND METHODS

### 2.1 Samples

In the present study, 70 gastric cancer tissues, surrounding adjacent gastric tissues and 25 benign lesions were selected from the department of gastroenterology surgery of affiliated Hospital of North Sichuan Medical College. All of the patients did not undergo radiotherapy or chemotherapy prior to the surgery. The adjacent cancer tissues were obtained at least 5 cm away from the tumor center and pathologically confirmed as normal gastric mucosa. The partial tissues were fixed with 4% formaldehyde and the rest was stored in liquid nitrogen for the following protein extraction and proteomics analysis. All of the experiments were approved by the Ethics Committee of North Sichuan Medical College.

### 2.2 Protein extraction

Thawed gastric cancer tissues (150 mg) were cut into pieces with scissors. RIPA lysis buffer and 10‐μl PMSF (Thermo Fisher Scientific) were added to the tissues. The sample was placed on ice and the suspension was treated by ultrasound (80 W, 15 s, 20 times), followed by centrifugation at 15,180 *g* for 15 min. The suspension was filtered by 0.22‐μm filter membrane and the filtrate was collected. The total protein concentration was determined using a Bradford protein assay kit (Bio‐Rad). The total protein samples were stored at −80°C.

### 2.3 iTRAQ labeling

The protein was processed by enzymolysis. Each 100‐μg protein was labeled with iTRAQ reagents for 2 h. The iTRAQ‐labeled samples were reconstituted in 4‐ml buffer A (25 mM NaH_2_PO_4_ in 25% ACN, pH 2.7) and loaded onto a 5‐μm particle size, 4.6 × 250 mm Ultremex SCX column (Phenomenex). The samples were eluted at a rate of 1 ml/min with a gradient consisting of 100% buffer A (25 mM NaH_2_PO_4_ in 25% ACN, pH 2.7) for 10 min, 5%‐35% buffer B (25 mM NaH_2_PO_4_, 1 M KCl, 25% ACN, pH 2.7) for 11 min, and 35%–80% buffer B for 1 min. the eluted peptides were pooled into 20 fractions. The peptides were processed to desalt and then evaporated to dryness using a SpeedVac.

### 2.4 LC‐ESI‐MS/MS analysis

The samples were resuspended in buffer A (2% CAN, 0.1% FA) and make the final concentration of peptide 0.25 μg/μl. With the LTQ Orbitraq Velos (Thermo) system, the sample volume was 10 μl per injection. Use a blank to clear the system after each sample. A data‐dependent procedure was applied to the MS scanning. The threshold ion count is 5,000. The mass spectrometer *m*/*z* scan range was 350–2,000 Da.

### 2.5 Database searches and bioinformatics

The identification of protein and relative iTRAQ quantification were performed with Mascot software (Version 2.2). Each MS/MS spectrum was searched against the International Protein Index (IPI) human database. The search parameters considered cysteine modification and biological modification. All of the proteins were grouped to minimize redundant. To estimate the false discovery rate (FDR), a decoy database search strategy was adopted for peptide identification. Correspondingly, a randomized database was generated. The data obtained above were then exported into Excel for manual data interpretation. A 1.2‐fold change threshold for all iTRAQ ratios was adopted to identify differentially expressed proteins between gastric cancer and adjacent tissue. The Blast 2 GO software and Kyoto Encyclopedia of Genes and Genomes (KEGG) database were used to perform ontology analysis and identify the tumor‐associated pathways with the differently expressed proteins.

### 2.6 Immunohistochemistry (IHC)

The paraffin‐embedded gastric cancer tissues were cut into 4‐μm thickness sections. The sections were then deparaffinized in xylene and dehydrated in ethanol. The sections were processed to antigen retrieval by boiling in 1 mM Tris‐EDTA (pH 9). The 6‐phosphogluconolactonase (PGLS) monoclonal antibody was purchased from Abcam. The sections were incubated with anti‐PGLS primary antibody (dilution factor 1:200) for 1 h at room temperature. The sections were then washed and developed with the DAKO REAL EnVision detection system (DAKO). The sections were incubated with HRP‐conjugated goats anti‐rabbits secondary antibodies for half an hour and then processed to DAB stain for 15 min. The sections were counterstained with hematoxylin for 30 sec and then washed, dehydrated in ethanol and xylene. The intensity and percentage of IHC staining were independently evaluated by two sophisticated pathologists without prior knowledge of clinicopathological information. The expression grade of PGLS was evaluated according to the staining intensity: negative (0), weak (+1), moderate (+2), strong (+3). The percentage of stained cancer cells was scored as follows: 0 (<5%), 1 (6%–25%), 2 (26%–50%), 3 (51%–75%), and 4 (76%–100%). The resulting scores were calculated according to the staining intensity scores and the scores of the percentage of stained cancer cells.

### 2.7 Oncomine database analysis

Oncomine database (https://www.oncomine.org/resource/login.html) includes 715 datasets and 86,733 samples (Research Edition). The mRNA expression level of PGLS gene in gastric cancer and normal tissues was analyzed according to the following parameters, data type of mRNA, gene rank of all, fold change of 1.5, and *p* value of ≦ 0.05. The study of D’Errico was selected to analyze the expression of PGLS genes in gastric cancer.

### 2.8 Kaplan‐Meier plotter database analysis

The Kaplan‐Meier plotter database (http://kmplot.com/analysis/) could assess the effect of 54,675 genes on patient's survival among 21 cancer types with the largest datasets, including lung cancer (*n* = 3,452), breast cancer (*n* = 7,830), ovarian cancer (*n* = 2,190), and gastric cancer (*n* = 1,440). The data mainly include gene chip and RNA‐Seq data from Affymetrix microarrays. By using Kaplan‐Meier plotter analysis tools, the effects of PGLS expression on gastric cancer patient's survival were assessed with a follow‐up time of 120 months for overall survival (OS) and post‐progression survival (PPS). The hazard ratio (HR) with 95% confidence intervals and log‐rank *p* values were also computed.

### 2.9 Statistical analysis

The fold changes, ranks, and *P* values were analyzed and displayed on Oncomine database analysis. The survival curves were analyzed by Kaplan‐Meier plotter database, with the HR, 95% CI and *p* values were also calculated. The correlations of PGLS expression with clinical parameters were calculated with Spearman's correlation and statistics significance. Chi‐square test was used to analyze enumeration data. Statistical analysis was performed with SPSS 22.0 (SPSS) for windows.

## 3 RESULTS

### 3.1 PGLS was found to be up‐regulated in gastric cancer

**FIGURE 1:**
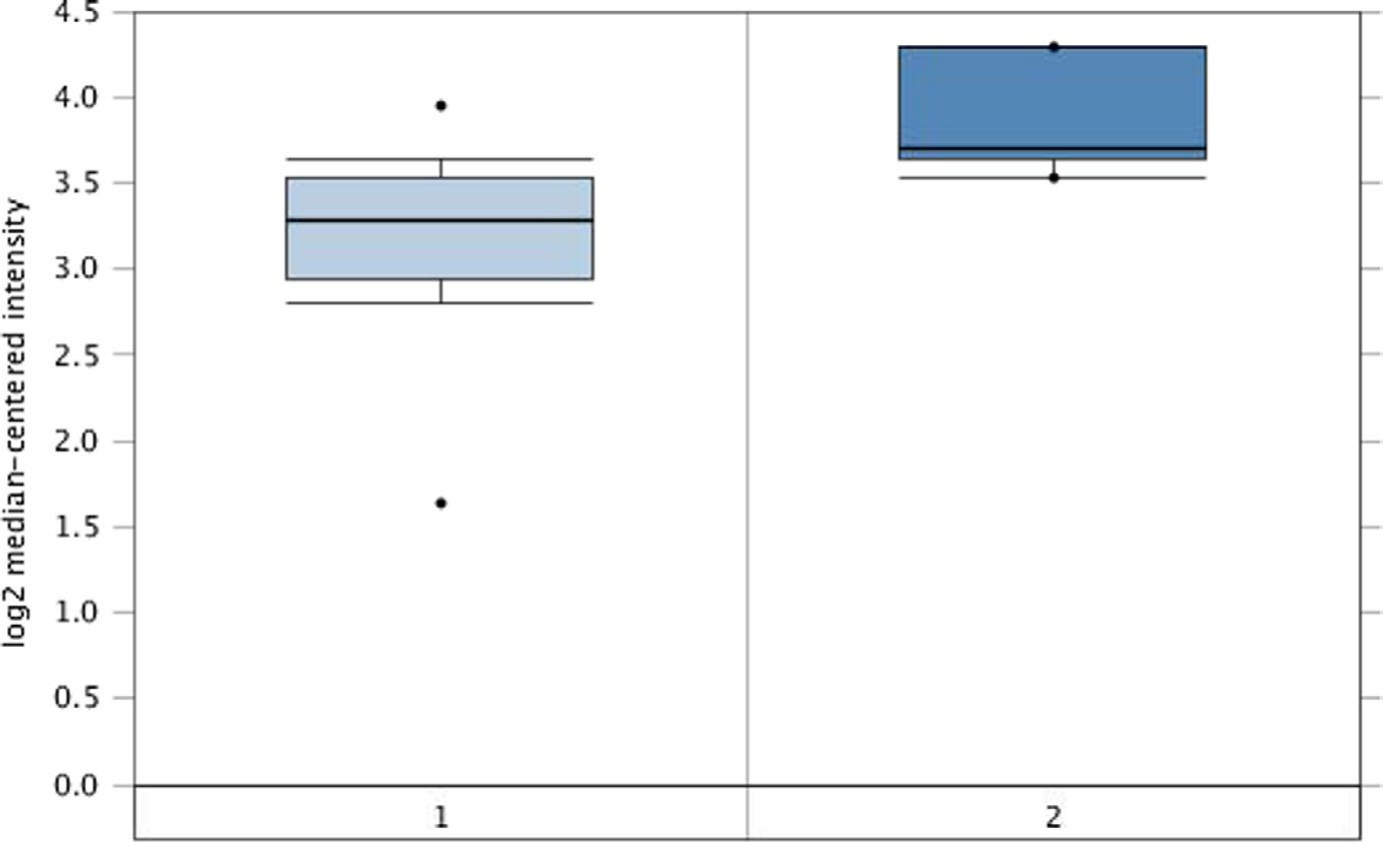
PGLS was increased expressed in gastric cancer compared with that in gastric mucosa with fold change of 1.523 from Oncomine database. *p *= 0.013. (1, Gastric mucosa; 2, Gastric mixed adenocarcinoma;)

The present study identified 431 proteins which were aberrantly expressed in gastric cancer including 224 proteins and 207 proteins that were increased and decreased expressed in gastric cancer tissues, respectively.^13^ Compared with the surrounding normal gastric tissues, PGLS was up‐regulated with 1.379‐fold changes in gastric cancer tissues. Moreover, from Oncomine database analysis, PGLS was overexpressed in gastric cancer compared with that in normal gastric mucosa with 1.523‐fold change (*p *= 0.013) (Figure 1).

### 3.2 Immunohistochemistry analysis of PGLS expression in gastric cancer

**FIGURE 2:**
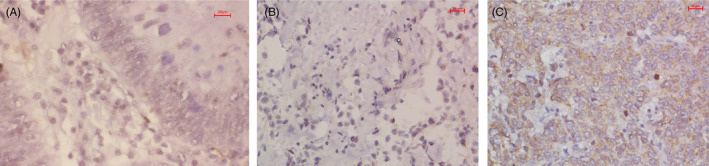
Immunohistochemistry study of the expression of PGLS in gastric cancer tissues. PGLS was mainly located to cytoplasm (A, Benign lesions; B, Adjacent tissues; C, Gastric cancer tissues)

Immunohistochemistry was performed to determine PGLS expression in gastric cancer tissues, adjacent tissues, and in benign lesions tissues. The results of immunohistochemistry showed that PGLS was strongly expressed in gastric cancer tissues compared with those in adjacent tissues (Figure 2). Among the 70 cases of gastric cancer, 51 (72.9%) cases presented strong positivity for PGLS. Of the 70 cases of adjacent tissues, 36 (51.4%) cases showed positivity for PGLS, while in benign lesions tissues, only 9 (36%) cases were stained by PGLS. The results are shown in Table 1.

**TABLE 1:** Immunohistochemical analysis of PGLS expression in gastric cancer, adjacent tissues, and benign lesions

Histological types	Cancer tissues	Adjacent tissues	Benign lesions	*p*‐Values
PGLS(+)	51	36	9	
PGLS(−)	19	34	16	0.002

Note

Chi‐square test was used to calculate the difference, with the significant level of *p *≤ 0.05.

### 3.3 Different pathological background between PGLS positive and negative expression patients

Table 2 shows the different pathological background parameters of PGLS‐positive expression and negative expression patients. To find out whether PGLS expression associated with patient's pathological background, such as histological type, T staging, lymph nodal positive status, and distant metastasis as well, we analyzed the pathological background of patients who had PGLS expression with patients who had not PGLS expression. Results showed that T staging (*p *= 0.02) was significantly associated with PGLS‐positive expression in gastric cancer patients. PGLS was significantly highly expressed in T3 and T4 stage patients compared with that in T1 and T2 stage patients (*p *= 0.02).

**TABLE 2:** Analyzing the correlations of PGLS expression with TNM staging variables in gastric cancer

Variables	PGLS(−)	PGLS (+)	Case	*p*‐Values
Histology classification				
Adenocarcinoma	15	45	60	0.323
Signet‐ring cell carcinoma	4	6	10	
pT				
T1 + T2	10	12	22	0.02
T3 + T4	9	39	48	
pN				
pN0	6	22	28	0.483
pN1 + pN2	8	14	22	
pN3a + pN3b	5	15	20	
pM				
pM0	19	50	69	0.539
pM1	0	1	1	

### 3.4 Clinicopathologic parameters correlated with PGLS in gastric tissues by IHC study

To further analyze whether the PGLS expression correlated with clinicopathological variables, we divided the subjects into several groups according to their clinicopathological variables. Chi‐square test was used to check the difference between subjects with PGLS‐positive expression and those without PGLS expression, with the significant level of *p *≤ 0.05. Table 3 shows the correlation of PGLS expression with various clinicopathological characteristics in gastric tissues. The results showed that the PGLS expression was significantly differed between gastric cancer patients with TNM Ⅰ staging and those with TNM Ⅱ, Ⅲ, and Ⅳ staging (*p *= 0.02).

**TABLE 3:** Correlations of PGLS expression with clinicopathological variables in gastric cancer

Clinicopathological variables	PGLS (−)	PGLS (+)	Cases	*p*‐Values
Age(year)				
≥60	12	34	46	0.783
≤59	7	17	24	
Sex				
Male	11	34	45	0.496
Female	8	17	25	
Histology classification				
Adenocarcinoma	15	45	60	0.323
Signet‐ring cell carcinoma	4	6	10	
Histological grade				
I	16	42	58	0.854
II and III	3	9	12	
Lymph node metastasis				
Positive	10	32	42	0.442
Negative	9	19	28	
TNM Staging				
I	9	10	19	0.02
Ⅱ‐Ⅳ	10	41	51	

Note

The significant difference was conducted with Chi‐square test, with the significant level of *p *≤ 0.05.

### 3.5 The PGLS expression and clinic prognosis of gastric cancer

**FIGURE 3:**
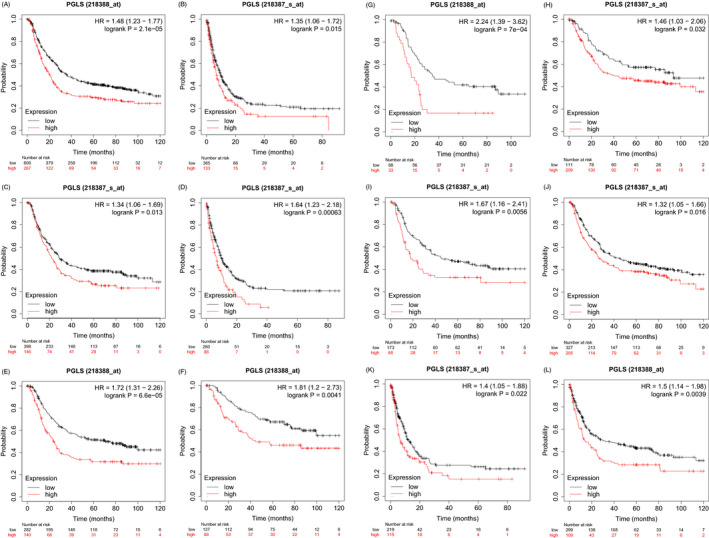
Kaplan‐Meier survival curves comparing the patients with high PGLS expression with those with low expression in different subgroups of gastric cancer. (A and B) Survival curves of OS, PPS in gastric cancer datasets in Kaplan‐Meier database (GSE14210; GSE15459. GSE22377; GSE29272; GSE51105; GSE62254; OS, *n* = 875; PPS, *n* = 498). (C and D) The PGLS high expression predicts poor OS (*n* = 544) and PPS (*n* = 348) in male patients with gastric cancer. (E–G) In patients with lymph node metastasis, high PGLS expression showed lower OS than those with low expression (*n* = 422 for all patients with lymph node metastasis; *n* = 225 for stage Ⅰ; *n* = 121 for stage Ⅱ). (H and I) The PGLS high expression predicts poor OS for patients with Lauren Intestinal type (*n* = 320) and diffused type (*n* = 241); (J–L) In the Her‐2 (‐) patients, PGLS high expression predicts poor OS (*n* = 532), PPS (*n* = 334), and FP (*n* = 408)

The potential prognostic values of PGLS in gastric cancer were analyzed through Kaplan‐Meier plotter database. Figure 3A, B shows that higher expression of PGLS predicts poor prognosis among gastric cancer patients (OS HR = 1.48, *p *= 2.1e‐05; PPS HR = 1.35, *p *= 0.015). Moreover, in male subjects, the high expression of PGLS predicts poor prognosis (OS HR = 1.34, *p *= 0.013; PPS HR = 1.64, *p *< 0.0001) (Figure 3C, D), while in female subjects, the PGLS expression was not significantly correlated with prognosis. Notably, the medium overall survival among gastric cancer patients with high expression of PGLS was 15.67 months shorter than those patients with lower expression of PGLS (*p *= 2.1e‐05). The mOS of male gastric cancer patients with high expression of PGS were 7 months shorter than those with lower expression of PGLS.

### 3.6 The PGLS expression predicts the poor prognosis of gastric cancer patients with lymph node metastasis

To further assess whether the PGLS expression impacts on prognostic values in gastric cancer patients with lymph node, we found that the high expression of PGLS predicts poor OS compared with those with low expression of PGLS among all patients with lymph node metastasis (HR = 1.72, *p *= 6.6e‐05) (Figure 3E). In detail, the PGLS high expression predicts poor OS in patients with stage 1 lymph node metastasis (HR = 1.81, *p *= 0.004) (Figure 3F), in patients with stage 2 (HR = 2.24, *p *= 7e‐04) (Figure 3G). Except for lymph node metastasis, in gastric cancer patients of Lauren intestinal type, the high expression of PGLS predicts shorter OS, with 53.1 months (mOS) less than those with PGLS low expression (Figure 3H, HR = 1.46, *p *= 0.032). In patients with Lauren diffused type, PGLS was also significantly correlated with poor OS showing 27.27 months (mOS) shorter than those patients with PGLS low expression (Figure 3I, HR = 1.67, *p *= 0.005).

### 3.7 The PGLS high expression predicts poor OS in patients with Her‐2 (‐)

In patients with Her‐2(‐), the high PGLS expression showed shorter OS and PPS than those with low PGLS expression (OS HR = 1.32, *p *= 0.016; PPS HR = 1.4, *p *= 0.022) (Figure 3J, K). The mOS of patients with PGLS high expression was significantly 18.9 months shorter than those with low expression in patients with Her‐2 (‐). The mPPS of patients with PGLS high expression was significantly 5.7 months shorter than those patients with PGLS low expression. Besides, the high PGLS expression was significantly correlated with shorter FP in patients with Her‐2 (‐) (HR = 1.5; *p *= 0.0039) (Figure 3L). The mFP of patients with PGLS high expression was significantly 16.27 months shorter than those with PGLS low expression. However, in patients with Her‐2 (+), the PGLS expression did not correlate with the OS, PPS, and FP as well.

## 4 DISCUSSION

In the present iTRAQ work scheme, a total of 431 proteins were identified in gastric cancer tissues. Bioinformatics analysis showed that those aberrantly expressed proteins in gastric cancer tissues were related to cell proliferation, differentiation, cellular movement, and cell death. Several proteins have been used earlier to predict cancer development and metastasis.^14^ In the present study, we found that PGLS was significantly highly expressed in gastric cancer tissues compared with that in adjacent gastric tissues. Further analysis indicated that PGLS may play an important role in the progression and prognosis of gastric cancer.

Metabolic reprogramming has aroused increasing attention. Metabolism is tightly regulated for nutrient uptake and metabolism in normal cells. Due to the acquired oncogenic mutations, cancer cells, however, could rewire the metabolic pathways to support its proliferation. In normal cells, pentose phosphate pathway (PPP) has fundamental functions which were essential for cell nutrients metabolism and macromolecular biosynthesis.^15^, ^16^, ^17^, ^18^, ^19^ First of all, PPP pathway generates ribulose‐5‐phosphate for nucleotide biosynthesis. Seconds, PPP pathway generates numerous NADPH, which is involved in macromolecular synthesis. Third, NADPH helps maintain reduced glutathione which is an important antioxidant that neutralizes free radicals in the body. Recently study has demonstrated that pentose phosphate pathway flux was up‐regulated in cancer cells.^20^

6‐phosphogluconolactonase, the key enzyme of pentose phosphate pathway, is to hydrolyze 6‐phospho‐δ‐gluconolactone to generate 6‐phosphogluconate (6PG). 6PG is proceeded to be dehydrogenated by 6‐phosphogluconate dehydrogenase (6PGDH) to generate ribulose‐5‐phosphate, which serve as the substrate for nucleotide biosynthesis. Recently, studies showed that PGLS was aberrantly highly expressed in various cancers, including cervical cancer, breast cancer, glioblastomas, and hepatic cancer, etc.^21^, ^22^, ^23^, ^24^, ^25^ Batsios et al found that PGLS was up‐regulated in glioblastoma and knockdown of PGLS could significantly reduce NADPH and GSH. By contrast, elevated PGLS produced steady‐state NADPH and GSH in vivo. Moreover, by detecting hyperpolarized‐[1‐^13^C] gluconolactone to form [1‐^13^C] 6PG, they could significantly differentiate the glioblastoma tumor tissues from normal brain tissues in vivo.^20^

To explore the site‐based differentiate expressed proteins in PPP pathway, Cha et al found that 6PGL (PGLS) was positively expressed in bone metastasis, with a shorter overall survival rates for breast cancer patients.^21^ Further, to explore potential cellular targets for mycoepoxydiene in cervical cancer, Jin found that PGLS was significantly down‐regulated after treatment of mycoepoxydiene, suggesting that PGLS is a potential molecular target of mycoepoxydiene for treatment of cervical cancer.^22^

In the present study, we found that PGLS expression was significantly higher in gastric cancer tissues than that of PGLS in adjusted cancer tissues. Further analysis found that the PGLS positivity in cancer tissues with T3 and T4 staging was significantly higher than that of patients with T1 and T2 staging, which indicated that the PGLS expression has strong impact on tumor stating. Moreover, we found that PGLS expression was strongly correlated with TNM staging Ⅱ‐Ⅳ, suggesting the poor prognosis of gastric cancer. Consistent with Ou's study, by using the methods of integrated proteomics, they found that 6PGL was strongly expressed in breast cancer tissues, while it was lowly expressed in all the normal breast tissues.^23^

Both in normal cells and tumor cells, PPP is the basic metabolic pathway. It produces ribulose‐5‐phosphates for nucleotide biosynthesis and reducing power in the form of NADPH that is needed for macromolecular biosynthesis and redox maintenance.^26^, ^27^, ^28^, ^29^, ^30^, ^31^ Many studies have targeted on glucose‐6‐phosphate dehydrogenase and 6‐phosphogluconate dehydrogenase for therapeutic treatments. However, the function of PGLS was not well studied partially due to the unstable property of 6‐phospho‐δ‐gluconolactone and rapid hydrolyzed to 6‐phosphogluconate. Recently, Gao et al demonstrated that knockdown of PGLS increased γ‐6‐phosphogluconolactone (γ‐6PGL) level in cell. The increased γ‐6PGL binds to Src and further enhanced PP2A recruitment, which ultimately inhibited the phosphorylation of AMPK and contributed tumor progression. This study provides new important evidence of PGLS in cancer development.^24^

Choi et al.^25^ detected the proteins of pentose phosphate pathway in breast cancer. They found that G6PDH, PGLS, and 6PGDH were significantly increased in breast cancer. Notably, the expression of PGLS was higher in Her‐2 (‐) breast cancer. They also found that PGLS was significantly associated with tumor staging, suggesting that PGLS may predict poor prognosis of gastric cancer.

Consistent with Choi's study, we found that PGLS expression was significantly correlated with OS and PPS in gastric cancer patients. The medium overall survival among gastric cancer patients with high expression of PGLS was 15.67 months shorter than those patients with lower expression of PGLS. Further analysis suggested that male gastric cancer patients with the high expression of PGLS predict poor prognosis while not in female patients. In addition, we found that the high expression of PGLS predicts poor OS among patients with lymph node metastasis ((HR = 1.72, *p *= 6.6e‐05)). Moreover, we also found that in Her‐2 (‐) gastric cancer patients, the high PGLS expression predicts shorter OS and PPS than those with low PGLS expression (OS HR = 1.32, *p* = 0.016; PPS HR = 1.4, *p *= 0.022;). The mOS of patients with PGLS high expression was significantly 18.9 months shorter than those with low expression in patients with Her‐2 (‐). These results suggested the converse correlations of PGLS expression with Her‐2 expression in gastric cancer.

In the present study, with the methods of quantitative proteomics analysis, we found that PGLS was significantly overexpressed in patients with gastric cancer. The PGLS positivity was strongly associated with TNM staging and predicts poor prognosis of gastric cancer. The study provided new evidence that the PGLS might be a potential diagnostic and therapeutic target for gastric cancer.

## CONFLICT OF INTEREST

The authors declare that they have no conflict of interest.

## Data Availability

Data are available in the article.
